# Comparison between outcome of single dose of prophylactic antibiotic versus postoperative antibiotic in inguinal hernia surgery

**DOI:** 10.12669/pjms.38.5.5332

**Published:** 2022

**Authors:** Ahmad Masood, Abdur Rehman Arshad, Mahnoor Ashraf

**Affiliations:** 1 Dr. Ahmad Masood, MBBS, FCPSI, MO, Department of Surgery, Nishtar Medical University & Hospital, Multan, Pakistan; 2 Dr. Abdur Rehman Arshad, MBBS, FCPSI, MO, Department of Surgery, Nishtar Medical University & Hospital, Multan, Pakistan; 3 Dr. Mahmoor Ashraf, MBBS, FCPSI, HO, Department of Surgery, Nishtar Medical University & Hospital, Multan, Pakistan

**Keywords:** Prophylactic, Hernia, Mesh, Antibiotic, Inguinal

## Abstract

**Objectives:**

To compare the outcome prophylactic antibiotics and routine pre-surgical and post-surgical in terms of incidence of surgical site infection (SSI) and to explore the effect of various factors such as duration of surgery and patient characteristics (if any).

**Methods:**

A double-blinded prospective analysis of a total of 60 patients with the primary inguinal hernia was conducted from 24^th^ August 2020 to 24^th^ August 2021 at the Surgical Department of Nishtar Medical University & Hospital, Multan. The participants of the study were categorized into two groups such that 30 consecutive patients were placed in the study group who were administered with a single dose of prophylactic antibiotic 30 minutes before to mesh repair surgery and the remaining 30 patients were placed in the control group who were administered routine antibiotics pre and post-operatively. The effects in patients were observed till 30 days following surgery for any sign of infection. All the collected data were analyzed through SPSS (version 19).

**Results:**

The rate of infection in both groups was noted. The incidence of infection in the study group (13.3%) was higher as compared to the control group (10%). No patient underwent mesh removal and no significant difference in terms of post-operative complications was observed in the results of both groups.

**Conclusion:**

Both the treatments, prophylactic antibiotics and routine pre-surgical and post-surgical were equally effective. However, we recommend the administration of prophylactic since they are cost-effective and prevent bacterial drug resistance.

## INTRODUCTION

Abdominal wall hernia is a prevalent disorder with an occurrence rate as high as 1.7% in all age groups and 4% in those aged above 45 years. Almost 75% of cases of abdominal wall hernias are accounted to Inguinal hernias which are potentially life staking in 27% of male patients and 3% female patients.^1^ Inguinal hernias often appear as a protrusion in the inguinal region that disappears when a little pressure is applied or when the patient reclines. The patient feels little to mild pain that may worsen due to increased activity.^2^ Various surgeries can be opted to treat this condition such as laparoscopic repair, open mash repair, nylon darn, ice layered, and Lichtenstein mesh.

Among them, mesh hernioplasty is the most preferred treatment as it has proved to be effective in reducing the relapse rates to only 1-2%. Currently, the most famous surgical method used for open repair of inguinal hernia is Lichtenstein mesh hernioplasty.^3^ Whereas, groin hernioplasty is done mostly to restrain peritoneal swelling through the myopectineal orifices. There are two ways to repair the hernias: either anteriorly by opening the groin such that appropriate division of the structures in and surrounding inguinal canal is done to reach the innermost layer of Apo neurotic fascia, or posteriorly through an abdomen incision which directly exposes the hernia orifices on entry to the preperitoneal space.^4^

Traditionally, groin hernia repair is a clean wound operation for which antibiotic prophylaxis is not required, as there is little to no risk of occurrence of surgical site infection (SSI) (<1%).^5^ It is presently the most preferred method technique for the plastic reconstruction of the inguinal region. Even though it is categorized as a clean surgery, there is a 0-9% risk of wound infection.^6^

This study was designed to compare the outcome prophylactic antibodies and routine pre-surgical and post-surgical in terms of incidence of surgical site infection (SSI) and to explore the effect of various factors such as duration of surgery and patient characteristics (if any).

## METHODS

A prospective analysis of 60 consecutive patients was conducted from 24^th^ August 2020 to 24^th^ August 2021 at the Department of Surgery of Nishtar Medical University & Hospital Multan after getting approval ERC Ref. No 90/148 dated 27-07-2020 from the hospital ethical committee. The patients diagnosed with a primary unilateral or bilateral inguinal hernia at the surgical OPD were consecutively included in the study such that the initial 30 patients were placed in the study group while the later 30 were part of the control group. The patients less than 18 years age; with recurring, incarcerated, strangulated, bilateral, or femoral hernias; diabetic patients, patients with liver or renal impairment; patients under steroid medication; antibiotic allergic patients; patients administered with antibiotics less than a week prior to surgery; patients with the impaired immune system; patients with local skin infections or disease at site of incision; pregnant or lactating patients were excluded from the study.

In the study group, patients were administered prophylactic antibiotics, injection ceftriaxone 1gm, 30 minutes prior to the surgery, and this group was not given antibiotics after the surgery. Whereas the patients in the control group were administered both presurgical and postsurgical routine IV antibiotics, ceftriaxone 1 gm every 12 hour. Both the groups were surgically treated with inguinal mesh repair.

Baseline data including demographics, type of administered anesthesia, surgery type, time of surgery, antiseptic used for disinfecting the skin and all infectious complications was maintained prior to the surgery. All the patients gave their informed consent to become a part of the study and their data was kept anonymous. Patients were free to terminate their participation in the study according to their discretion whenever they like, given that their right to treatment is not compromised.

All the patients in both groups underwent a standard Lichtenstein hernia repair. Skin preparation of all patients before the surgery was done with Povidone-iodine as an antiseptic. Groins were shaved on the day before surgery. After the surgery, a standard sterile dressing was used for post-operative wound management. No postoperative antibiotics were administered to study group. Forty-eight hours after the surgery, the wound was inspected and the dressings were removed. The dressing was not applied afterward.

Surgeons who did not perform the surgery did a postoperative follow-up of the cases at 7^th^ day and after one month, post-surgically. Wound was inspected for any sign of discharge, discomfort, or redness. If these conditions were observed, they continued following the case for up to one month. According to definitions provided by disease control centers, wound infection can be of two types; superficial surgical site infection (SSI) and deep surgical site infection (DSSI). The primary endpoint of the study was wound infection, according to the criterion defined by ASEPSIS and criteria:

Patients were diagnosed with Superficial SSI under the following criteria: Development of infection within one month following the surgery such that only subcutaneous tissue and skin of the incision are involved and patient has at least one of the following:

- Pus discharge from the site of incision.
- Microorganism isolated in cultures of aseptically collected tissue or fluid from the superficial incision.
- A culture-positive superficial incision with one of the following signs and symptoms of infection: redness; localized swelling; heat; or pain.

### The patients were diagnosed with Deep SSI under the following criterion:

Development of infection within one or three months after the surgery such that soft tissues, facial and muscle layers, of the incision are involved and the patient has at least one of the following:

- Pus discharge from the site of incision.
- A culture-positive deep incision, deliberately opened by the surgeon, with one of the following signs and symptoms of infection: fever more than 38 °C; tenderness or localized pain.
- Discharge of pus or any other sign of deep-site infection evident on direct examination, invasive intervention, imaging studies, or histopathological testing.

The patients were thoroughly examined to eliminate SSI. Initially, infections were managed with dressings merely; however, if required, the discharge was let out by removing sutures. If the patient showed no improvement or the infection started increasing, antibiotics were administered. The results were evaluated with regards to superficial and deep surgical site infection (SSI).

### Statistical Analysis:

SPSS software to analyze all the patients’ data. Non-parametric data were analyzed using the Chi-square test and parametric data were evaluated using Fisher exact test and the Student t-test.

## RESULTS

Both the groups (study & control) consisted of 30 patients. The patients in both groups were compared on basis of baseline characteristics including age, gender, type & side of the hernia. The average age was 35.66 years and 37.25 years in the antibiotic group the control group respectively, with the range of patients being from 18 to 70 years.

Out of 30 patients in the study group, 18 patients (60.0%) had a hernia on the right side and 12 patients (40.0%) had a hernia on the left side. In the control group (30 patients), 16 patients (53.3%) had a hernia on the right side while 14 patients (46.6%) had a hernia on the left side. With regards to the type of hernia, 24 patients (80.0%) had a direct hernia and six patients (20.0%) had an indirect hernia in the antibiotic group. In the control group, 19 patients (63.3%) had direct hernia and 11 patients (36.6%) had an indirect hernia. A total of 43 patients (71.6%) and 17 patients (28.3%) had direct and indirect hernias respectively. The patients’ demographic data are shown in Table-I.

**Table I:** Patients’ demographic data.

		Study group (n=30)	Control group (n=30)	Total
Age (years)	Mean value	35.66 years	37.25 years	Range 18 to 70 years
Site of hernia N (%)	Right	18 (60)	16 (53.3)	34 (56.6)
	Left	12 (40)	14 (46.6)	26 (43.3)
Type of hernia N (%)	Direct	24 (80)	19(63.3)	43 (71.6)
	Indirect	6 (20)	11 (36.6)	17 (28.3)

Both the groups were analyzed for the occurrence of infection. At the time of discharge, one patient (3.33%) in each group was infected (p>0.05). A follow-up was done at the time of suture removal which revealed that no patient was infected in both groups. Fourteen days after discharge, 2(6.66%) patients in the study group and only one patient (3.33%) was infected in the control group. The difference between the rate of infection after seven days and 14 days of discharge was not statistically significant (p>0.05). The occurrence of infection was again followed up after one month of discharge. One patient (3.33) in both groups showed evidence of infection. The difference between the infection rate in both groups was not statistically significant (p>0.05). None of the patients developed deep SSI and also mesh removal was not required in any patient due to SSI. (Table-II).

**Table II:** Follow up of wound infection in both groups.

	Study group (N=30)		Control group (N=30)		(n=60)

Time after surgery	Infection present N (%)	Infection absent N (%)	Infection present N (%)	Infection absent N (%)	Total infected N (%)
At discharge	1 (3.33)	29 (96.6)	1 (3.33)	29 (96.6)	2(3.33)
At suture removal, one week after discharge	0(0)	30 (100)	0(0)	30 (100)	0
Two weeks after discharge	2 (6.66)	28 (93.3)	1 (3.33)	29 (96.6)	3(5.0)
One month after discharge	1 (3.33)	29 (96.6)	1 (3.33)	29 (96.6)	2(3.33)

The overall occurrence of infection was 11.6%. Out of which, 13.3% and 10% rate of infection was found in the study group and control group, respectively. Though the number of patients infected study group were more than in the control group, the infection rate was statistically insignificant (p<0.05).

The wound site was tested for microorganisms and it was observed that out of seven patients with wound infection, four patients were detected having staphylococcus infection, four having streptococcus, and only one patient was detected of infected with Klebsiella. All the patients diagnosed of streptococcus infection were analyzed one month after the discharge and were investigated if infected with a secondary infection.

Both the groups were also analyzed for any post-operative complications. Only one patient in the control group developed urinary retention after the surgery. The patient was catheterized and discharged. De-catheterization was successfully done at the time of suture removal.

One patient in the control group was also diagnosed with hydrocele and was treated conservatively till 1-month post-surgery. The patient was analyzed 30 days after the discharge and was advised to visit again for hydrocele surgery after six months but he did not join the follow-up. The complication rate of both groups was statistically insignificant (p>0.05).

**Table III:** Isolated microorganisms cultured in patients with infections.

Microorganism	Total
Staphylococcus	4 (57.1)
Streptococcus	2 (28.6)
Klebsiella	1 (14.3)

## DISCUSSION

Over one million patients in the USA and Europe undergo inguinal hernias repairs every year. Yerde et al performed the first randomized control trial in 2001 to study the effects of antibiotic prophylaxis in mesh repair of inguinal hernia. The study supported the use of prophylactic antibiotics.^7^

A cochrane meta-analysis was conducted in 2004 about the prophylactic use of antibiotics but the study’s result remained indecisive about its use. Inguinal hernia repair is one of the most frequently used operative methods all over the world; therefore, both misuse of antibiotics and a high rate of surgical site infection incurs high medical and social expenses. So, it is important to have a piece of definite evidence about the use of antibiotics.^8^,^9^

The opponents of antibiotic use argue that the patients undergoing Lichtenstein hernia repair still develop infection even after antibiotic administration and when overused, it can lead to antibiotic resistance. Moreover, it is claimed the patients could develop the risk of fatal allergic reactions, and since many patients undergo this procedure, a greater strain is put on the health budget with regular antibiotic use.

On the other hand, the occurrence of infection after the mesh repair increases its recurrence by four folds and may require drainage and mesh removal. This indicates that mesh does not pose a risk of infection but when the infection develops it is severe.^10^

**Table IV:** Complications in patients of both groups.

Complications	Antibiotics group (n=30)	Control group (n=30)
Urinary retention	0(0)	1(3.33)
Hydrocele	0(0)	1(3.33)
Seroma formation	1(3.33)	1(3.33)
Orchitis	0(0)	0(0)

Tzovaras et al conducted a study to determine the role of antibiotic prophylaxis in elective open inguinal hernia repair with a prosthetic mesh.^11^ It was revealed by their study that antibiotic prophylaxis did not have any significant benefit in this procedure. The same conclusion was drawn from a randomized prospective study of Al-Fatah et al.^12^ On the contrary, Ullah et al, conducted a study on 166 patients, dividing them into antibiotic and placebo groups, and concluded that antibiotic prophylaxis is a more effective treatment for mesh plasty.^13^

Several studies have been conducted to test the efficacy of antibiotic prophylaxis in elective hernia repair but the conclusions are different in all of them due to differences in study conditions.

The occurrence rate of SSI after mesh repair of inguinal hernia is 0-9%. The total rate of infection in our study was higher than this rate 11% (13.3% and 10% in the study group and control group respectively) due to the limited number of patients and type I error.

In the study conducted by Yerdel et al.^7^, the rate of infection was 9% and 0.7 in the control group and antibiotic group respectively. The study by Celdran et al and Usang et al also drew the same conclusions from their research as the incidence of SSI in the control group was more than that in the antibiotic group.^14^,^15^

Tzovaras et al, Aufenacker et al, and Perez et al concluded from their study that the prophylactic antibiotic did not prevent SSI after inguinal mesh repair.^11^,^16^,^17^ Total 85% of the patients in our study were infected with superficial SSI. Similarly, in studies by Tzovaras et al, Celdran et al, Ergul et al, Jain et al, all the SSIs were superficial SSI.^11^,^14^,^18^,^19^

According to research, the prevalence of mesh infection reported ranges from 0.35-1%. None of the patients in our study had deep SSI and hence no mesh removal. Similarly, no patients in the Aufenacker et al and Othman had mesh removal.^16^,^20^ One patient in each group in Perez et al,^17^ one patient in Shankar et al,^21^ and three patients in the placebo group with DISSI in Yerdel et al^7^ had subsequent mesh removal.

The recurrence rate in SSI and DSSI should be analyzed. According to a study by Celdran,^14^ which was conducted using a prosthesis, an increase in the rate of incidence of infection does not increase the rate of recurrence. In the case of mesh removal too, the fibrotic reaction surrounding the posterior wall of the inguinal canal can prevent the recurrence. The rate of recurrence in our study was 0% but it could be due to shorter follow up period.

In order to achieve a 50% difference between both the groups and have significant statistical power, the study conducted should be multi-center with at least have 800 patients in each group.

### Limitations:

Our study power is small due to a smaller number of patients and single-centered study and this is a limitation of our study.

## CONCLUSION

Both the treatments, prophylactic antibodies and routine pre-surgical and post-surgical were equally effective. However, we recommend the administration of prophylactic since they are cost-effective and prevent bacterial drug resistance.

### Authors’ Contribution:

**AM & MA:** Conceived, designed and did statistical analysis & editing of manuscript.

**ARA & MA:** Did data collection and manuscript writing.

**AM & MA:** Did review and final approval of manuscript.

**AM:** Responsibility for the accuracy & integrity of the study.
